# Criticisms on the Treatment of the Venereal Disease

**Published:** 1802-07

**Authors:** T. Vage


### *Criticisms on the Treatment of the Venereal Disease.*

By T. Vage, M. D. F. R S.

[ Continued from Vol. VII. p. 559—564. ]

THE nocturnal pains, &c. of the ſecond ſtage of the diſeaſe, are commonly very ſevere, and the mitigation of them, of of courfe, is an important concern; efpecially as the fpecific itfelf is incapable to procure eafe for a confiderable time. Opiates are ufually, and properly, given in this intention; but notwithftanding their utility, a free and frequent ufe of them always induces a relaxation of the fyftem, and debilitates the chylific organs, which are primary things to guard againft, in mercurial courfes. Although both thefe effects of opium appear to fpring from one common fource, by producing a nervous, fedative ftupefaction, yet fome obfervation in practice inclined me to fuppofe, that eafe may be procured without any concomitant debility. The eafe of opiates is the confequence of forced fleep; but fleep, inftead of being the caufe, is often the direct effect of eafe. All inflammations and ulcerations become more painful, it may be obferved, upon any febrile impulfe in the circulation, and eafier in proportion as that impulfe is diminifhed. The reafon of it is, that the nervous fibrillæ are, on extenfion, in a ftate of inflammation or corrofion, and any additional force in the circulation muft increafe the tenfion, and confequently the pain; as, by a parity of reafoning, every diminution of that force will produce relief. The digitalis is a fubftance well afcertained by the ingenious Dr. Maclean, and others, to have the property of retarding the circulation to a remarkable degree; and it was reafonable to imagine, that it would, upon that account, anfwer the prefent intention; and its good effects in phthifis is probably owing to the fufpenfion of irritation produced by this means, by which the pulmonary ulcerations receive time to heal. Upon this principle I have given the digitalis for fome time, and think it may be fairly recommended to the notice of others.

We fhall now beftow a few obfervations upon the infirmities of conftitution, which frequently remain after the difeafe has been perfectly eradicated, and after every atom of the mercury itfelf has been difcharged from the fyftem. Thus it is common to fee patients of this defcription, not only reduced to a moft debilitated ftate of both body and mind, but fubject, from the flighteft caufes, to a variety of other complaints, as rheumatifm, dropfy, afthma, dyfentery, and particularly typhus fever. Thefe infirmities are caufed, either by the difeafe itfelf, or by the remedies or methods of practice. The fubject, as a matter of difquifition, is fomewhat novel, but we fhall attempt an explanation of it. The defects of the conftitution, produced by the venereal poifon, always arife from the parts which were, in fome degree or other, ulcerated; for the parts which were only inflamed, or indurated, recover their healthy ftate as the infectious irritation fubfides. But in the ulcerated parts there is lefs of fubftance; fome of the fmall veffels, mufcular fibres, nerves, &c. are deftroyed, which, according to the importance of of the part, that is, the more they belong to the principal offices of the fyftem, the vital, natural, and animal functions, the more they will leave the habit in an impaired ftate. Hence it happens, that as fuch places are not within notice, confiderable blame is fometimes caft upon practice and practitioners without reafon. For though one mode of practice may ftop the further deftruction of organized parts fooner than another, yet it is evident, none can replace thofe already deftroyed. Mercury, however, with all its anti-venereal properties, is naturally inimical to the nervous fyftem, and exerts its injurious effects, in fome degree or other, in the moft judicious ufe of it. When it is exhibited too copioufly, and fuddenly, it is apt to produce violent effects, as great fwelling of the head and tongue, apoplexy, &c. becaufe it breaks down the blood before any outlet is prepared for its evacuation. When its ufe is gradual, thefe effects will be moderate, but they will accumulate in time to confiderable injuries of the fame nature. The moft violent and mildeft effects of remedies are produced upon the fame principle; and the former are frequently the only index to explain the latter, which would otherwife be two minute for obfervation.

The infirmities which arife from the ufe of mercury, appear to originate from two principal fources; one is, its diffolution of the blood, by which a redundance of ferum is forced into the interftices of the cellular fubftance of the mufcular, vafcular, and nervous fyftems, in confequence of which, the gluten* which gives ftrength and ftability to the folids, becomes relaxed, and the different functions of the animal economy fo debilitated, as to be incapable to be properly actuated by the nervous influence, while the nervous fyftem itfelf may remain in a tolerable condition. The other fource of infirmity, on the contrary, is when the nervous fyftem has been left impaired, and cannot invigorate thefe functions, which may not have fuffered any confiderable detriment. For it is experimentally afcertained, that if the nerves of any part are injured, either at their origin or in their courfe, that part will become proportionably inert in its office. The mode by which the nervous debility is here effected, appears abftrufe; it would feem to be the oppofite, or diminution of that influence, on which mufcular motion depends, and which has hitherto evaded demonftrative knowledge, becaufe moft of the functions of the fyftem are performed by mufcular fibres. It is certain, from many inftances, that fuch a general debility can be produced by the difeafed affections

* Vide Haller's Primæ Liniæ.

affections of the nerves alone; for it can be done before any change is wrought in the other parts of the body; and there are fome things which, even by a local application, can at once fuf-pend, or totally deftroy, the vital actions. The effects of mercury on the nervous fyftem, feem fomething fimilar to thofe of lead; both have power to produce paralytic affections; both, in a weaker degree, abate inflammations and mitigate pain; and the imbecility of both remain, after they have been quite expelled from the habit. But however abftrufe the nervous power may be, in its modes of action upon the folids, we have one thing in favour of our abilities, that it is, in a great meafure, conftrained to operate but in a two-fold manner, either by invigoration or weaknefs; and experiment and obfervation have difcovered a multiplicity of things, which act in thefe refpects with quicknefs and energy, and which would feem, if methodically and clofely regarded, to reflect an additional light on the nature and cure of difeafes. I have been accuftomed to arrange fuch things under three general heads, enervants, irritants, and cardiacs. The two laft are ufually, but erroneoufly, confidered under the idea of ftimulants. Both, indeed, effect invigoration, but they produce it in a very different way. Irritants imply a degree of pungency and pain, as muftard, horfe-radifh, volatile falts, &c. but give no refection, nor any nutritious ftrength: while cardiacs, fuch as wine, fpirits, the odours of fome plants, &c. are, in a great degree, congenial to the nervous fyftem, and may be confidered as immediate fubftitutes for what is prepared in the brain, when the ftrength is exhaufted by ficknefs or fatigue. Enervants, as the word imports, have properties to debilitate or reprefs the nervous influence; yet many things, which agree in this refpect, differ widely in others; as opium, digitalis, preparations of lead, lauro-cerafus, &c. and mercury, according to our prefent hypothefis. The effects of enervants appear hurtful, chiefly when the nervous influence is at or below its natural ftandard; when it is above it, they become remedial by their fedative efficacy. Irritants and cardiacs are ufeful, or hurtful, where the former have contrary effects; that is, ufeful in cafes where the fenfibility of the fyftem is on the ebb, and hurtful where it is too fufceptible and energetic.

The nervous fource of mercurial infirmity, and the humoral one, may indeed exift feparately, but they are generally combined; and their methods of treatment may be confidered as co-efficient, and united alfo. As the folids, and efpecially the alimentary vifcera, in thefe cafes, are in a relaxed condition, both the irritant and cardiac plans of invigoration muft be employed; and the firft thing indicated, is a fupply of light nourifhment, riſhment, as near as poſſible to its natural ſtate of aſſimilation. Young food is moſt eligible in this point of practice; for young animals are nouriſhed without the help of ſtrong digeſtive faculties; hence their ſuſtenance is eaſily aſſimilated, and ſuitable to their feeble ſtate, and conſequently their juices are fitteſt for weak or decayed conſtitutions. And agreeably to this opinion, it is obſervable, that theſe juices have naturally a diſpoſition to jelly, which they loſe as animals advance to maturity. Generous wines may be allowed at proper intervals, and in a moderation proportioned to the former habits of living. When the ſtomach is very languid, and inert, a glaſs of muſtard whey, at times, is a good nutritive irritant; and where there is much depreſſion of mind, which is ſometimes the caſe, when patients have been addicted to ſpirituous liquors, a little French brandy may be added to the whey. Beſides liquid aliments, ſome light ſolid food, however, ought never to be omitted; as by maſtication, it promotes the ſecretion of the ſaliva, one of the digeſtive fluids, and is afterwards a natural ſtimulant for the action of the inteſtinal canal. The obſtinacy of theſe complaints, frequently obliged me to deviate from common doctrines; and many practical reaſons induced me to obſerve, that the nearer animal juices are to their natural, commonly called their crude ſtate, the eaſier they are of aſſimilation, and more nutritious; for by that degree of heat, which is uſually employed to extract them, they ſeem to undergo a change, which again requires the aſſiſtance of ſtrong digeſtive powers. But as animal ſubſtances are apt to run into putrefaction in cold water, before they ſufficiently impregnate it, it was ſuppoſed, that if they were infuſed in wines, and other cordial liquids, which reſiſt the egetic proceſs, and are, at the ſame time, capable to extract the nutritious parts of them, conſiderable advantage would accrue. Agreeably to this idea, I was induced to try preparations of this nature, and found them to anſwer ſo well, that I have been in the habit of preſcribing them for ſome years paſt, not only in theſe caſes, but in conſtitutions broken from other cauſes, where nutritious reſtoratives were judged neceſſary, and wherever the digeſtive viſcera were in a dyspeptic condition. The parts of animal food moſt conducive to this end, ſeem to admit of a very judicious choice; for we find conſiderable difference in the texture, taſte, &c. between the different parts of the ſame animal, although they draw nutrition from the ſame common ſtock of digeſted aliment: and this can only ariſe from a peculiarity of fabric in each part, which ſeparates and aſſimilates further its own proper nouriſhment. According to this poſition, as ſingular as it may appear, is there not ſome reaſon reafon to infer, that preparations of fuch parts, in the manner above-mentioned, as having already undergone the changes neceffary for that peculiar fitnefs, would in fome meafure be applicable to fimilar parts in the human fyftem, when they are by any means impaired, or when the principal offices of digef-tion are debilitated? Vegetables, from their fuppofed flatu-lency in weak habits, are generally prohibited, but probably in the extreme. Animal food alone, has its difadvantages; a pro-per mixture of both is neceffary for falutary nutrition. By what means they qualify each other, cannot be here explained; but it is certain, that even fuch as are reckoned the moft fla-tulent, may be given in moft cafes, with advantage, in a fuita-ble proportion; and fome there are, fuch as water and garden creffes, and the like, which from their warm and irritant nature, are particularly ufeful. But this fubject fhall be more fully handled in a future Paper, when we come to the treat-ment of conftitutions enervated from other caufes, as luxury, irregularities, and intemperance.

The interftitial effufion is the next confideration; and though this does not always appear in dropfical fymptoms, yet it al-ways occupies the internal receffes of the fyftem, and is ufu-ally marked with a pallid flaccidity of countenance and fkin. The emunctorial evacuations, here, efpecially cuticular and renal, muft be promoted, for fome time, above their natural quantity, but without exhaufting, in any degree, the ftrength or fpirits; fo that, in this refpect, evacuants may be confidered as tonics and bracers: For by removing the redundant humi-dity, the fibres of the relaxed parts will recover their cohefive and contractile power. But cathartics, and particularly draftic ones, muft be avoided; the bowels are in a ftate of humid re-laxation already, and any confiderable irritation will bring an additional flux of humours, and increafe their debility; all that is neceffary in this point of practice, is only the aid of fome laxatives, occafionally, to keep up the energy of their vermi-cular motion. Whatever propriety there may be in active purgatives, immediately after a courfe of mercury, they are, certainly, injurious in the debility which is left (at leaft by common practice) upon the habit. But if, at any time, an ac-tive cathartic is given, the only way to derive benefit from it, and which, in common inflammations I have experienced, is an abftinence from liquids during its operation.

In the corroboration of the folids, the chylific vifcera claim the chief attention; fo much fo, that in other cafes of general debility as well as thefe, the invigoration of them may be re-garded as the re-eftablifhment of the whole: For on the pro-per digeftion of the food, depend thofe further changes that fit fit the chyle for the various offices of nutrition and health; our fluids become organized by it; they acquire a confiftency, and cohefion, which difqualify them for interftitial effufion, and which, at the fame time, fuftains that gluten, which gives ftability to the parts, and which is always relaxed when our humours are poor and watery. It avails but little to evacuate morbid fluids, if no better are fupplied.

All the functions of the animal economy, indeed, mutually affift each other in the recovery and confervation of health; but the natural ones may be regarded as the bafis of the reft: For before our aliment can be fit for nutrition, if of a vegetable nature, it muft be in fome degree animalized; or if an animal fubftance, it muft be affimilated to the particular conftitution it is to nourifh; and all this is effected by the digeftive organs, and a mixture with the fluids fecerned into them, which have already undergone thefe changes; fuch as the faliva, gaftric, and pancreatic, and hepatic fecretions. Whatever may be the particular ufe of thefe fluids in chylification, it is certain that the fuppreffion or deficiency of any one of them, will fenfibly hurt nutrition, and impair health. And here it may be ufeful to remark, that the three laft of thefe fecretions depend very much upon the due vermicular action of the inteftinal tube, and which, in thefe cafes, is generally inert. In this intention of cure, I found it advantageous to begin with an emetic, not merely for the evacuation of the ventricular contents, but for the extenfive agitation of the habit, which the irritation of the ftomach produces. The exhibition of warm bitters muft, afterwards, be adhered to; among which cinchona and gentian hold a chief rank, and which will be much improved by the addition of aromatic fpices. When the enervation of the ftomach is urgent, as may be known from flatulencies and eructations after food, with giddinefs, vertigo, and naufea, a moft efficacious bitter is a ftrong infufion of flor. chamomel. with fome elix. vitr. acid. This acid has a fingular property to arreft the fermentation of vegetable and animal fubftances, but its ufe muft be only occafional, from its tendency to obftruct the cuticular pores.

[ To be continued. ]

ACCOUNT

